# The Eighth Annual Bio-Ontologies Meeting

**DOI:** 10.1371/journal.pcbi.0010077

**Published:** 2005-12-30

**Authors:** Phillip Lord, Robert Stevens, James A Butler, Robin McEntire

## Scientific Mission

The annual Bio-Ontologies Meeting has been operating for eight years; during this time, it has stimulated discussion about the role of ontologies and their associated technologies for structuring, sharing, analysing, and searching knowledge about biological systems. The format varies; usually, it has a mixture of talks and structured discussions such as panel sessions. It regularly draws a hundred participants, showing a consistently high interest across the Intelligent Systems for Molecular Biology (ISMB) community.

The meetings were initially characterised by evangelism for the use of ontoloiges and by arguments about the nature of ontology and which representation was best. Now the debate centres on how to use and how to improve new ontologies.

## Hot Topics

Ontologies have an increasing presence in bioinformatics, particularly since the Gene Ontology Project demonstrated the value of supplementing genomic annotation with ontology terms. This interest from the biomedical sciences is intersecting with the interest from scientists and technologies in general. The Semantic Web vision now being actively promoted as the “next generation” Web—which aims to make the Web open to automatic computational use—makes heavy use of ontological technology (http://www.w3.org/2001/sw); this, in turn, is leading to increasing provision of mature tools and experience, lessening the activation energy for those wishing to develop or use ontologies.

One of the themes of this year's meeting, highlighted by Mark Musen's keynote address, are the attempts to move bio-ontology development to a much larger scale (further information about this talk and others mentioned in this article are available from the Bio-Ontologies Web site). Ontology development is often carried out by small groups of individuals, mirroring much of biology before high-throughput technologies. With the large numbers of ontologies now available (for examples, see http://obo.sourceforge.net), orthogonality, maintenance, and consistency are becoming key issues. Currently, there is a relatively poor understanding of both appropriate best practises and the technology that will be required to support these best practices: what, for instance, are the best mechanisms for peer review of ontologies; is centralised management necessary or are more decentralised approaches possible?

Many of the research talks also touched on these issues. Several people discussed applications of automated reasoning techniques: enabling complex querying over data from the yeast community [1] or moving towards the automation of protein classification as part of the process of genome annotation [2]. Ontologies are increasingly being used statistically, often to augment or refine experimental results—in the case of Vailaya et al. [3], this was microarray data. Several talks described new techniques for ontological engineering, new logics better able to describe change in biological systems [4], or more formal treatment of pathological anatomical features [5].

This year, the Bio-Ontologies Meeting was also able to hold a well-attended poster session. Several of the posters described new warehousing environments [6,7] or tools [8,9]. Finally, a number of posters described existing projects from several viewpoints [10,11,12].

Historically, the main use of ontologies within biology was to enable a de facto integration between different data sources by providing a common vocabulary. There are, however, now recognised to be many uses beyond the provision of vocabulary. The use of ontologies within bioinformatics started as complex schema for knowledge databases such as EcoCyc (http://www.ecocyc.org)—a use which continues to this day. The use of ontologies in data analysis is also becoming commonplace.

As with much of bioinformatics, we see a great interest in bio-ontologies from the computer science community. Bio-ontologies offer a variety of large, rapidly changing examples of ontologies with which knowledge representation techniques, methodologies, and tools can be developed and tested. At the Bio-Ontologies Meeting, this relationship has grown from mutual incomprehension to, by and large, a useful symbiosis.

## Breaking News 

### 

#### Pacific Symposium on Biocomputing 2006.

This year, the Pacific Symposium on Biocomputing will feature a new Semantic Web track, called Semantic Webs for Life Sciences. This includes a tutorial, a paper session, and panel/discussion session. The conference will be held on 3–7 January 2006 in Maui (http://psb.stanford.edu/cfp.html; http://www.cs.man.ac.uk/~stevensr/events.html).

#### ISMB 2006.

One of the most pleasant surprises about the 2005 Bio-Ontologies Meeting was the increase in the number of paper submissions, about 30 papers. We were lucky to be able to accommodate so much excellent work with the late introduction of a poster session. Given this increase in submissions, for next year, we hope to modify the publication process—we would like authors to have more space to explain their science than we have currently been able to provide.

Finally, we are currently investigating ways to interact better with other special interest groups. The programme at ISMB is now very full, with many different special interest groups providing excellent science, although with conflicting schedules. For 2006, we are investigating coordinating with BioLink; the synergy between ontological representation and natural language techniques is a natural one. This should ensure that attendees get maximal benefit from both programmes. We welcome any input (E-mail: bio-ont-sig@cs.man.ac.uk). 

## Abbreviation

ISMB: Intelligent Systems for Molecular Biology
